# Comparison of an open view autorefractor with an open view aberrometer in determining peripheral refraction in children

**DOI:** 10.1016/j.optom.2021.12.002

**Published:** 2022-01-10

**Authors:** Pelsin Demir, Antonio Filipe Macedo, Ranjay Chakraborty, Karthikeyan Baskaran

**Affiliations:** a Department of Medicine and Optometry, Linnaeus University, Kalmar, Sweden; b Center of Physics, Optometry and Vision Science, University of Minho, Braga, Portugal; c College of Nursing and Health Sciences, Optometry and Vision Science, Sturt North, Flinders University, GPO Box 2100, Adelaide, SA, 5001, Australia; d Caring Futures Institute, Flinders University, GPO Box 2100, Adelaide, SA, 5001, Australia

**Keywords:** peripheral refraction, autorefraction, aberrometer, refractive error, schoolchildren, myopia

## Abstract

**Purpose:**

The aim of this study was to compare central and peripheral refraction using an open view Shin-Nippon NVision-K 5001 autorefractor and an open view COAS-HD VR aberrometer in young children.

**Methods:**

Cycloplegic central and peripheral autorefraction was measured in the right eye of 123 children aged 8 to 16 years. Three measurements each were obtained with both Shin-Nippon NVision-K 5001 autorefractor and COAS-HD VR aberrometer along the horizontal visual field up to 30° (nasal and temporal) in 10° steps. The refraction from the autorefractor was compared with aberrometer refraction for pupil analysis diameters of 2.5-mm and 5.0-mm.

**Results:**

The Shin-Nippon was 0.30 D more hyperopic than COAS-HD VR at 2.5-mm pupil and 0.50 D more hyperopic than COAS-HD VR at 5-mm pupil for central refraction. For both pupil sizes, the 95% limits of agreement were approximately 0.50 D for central refraction, and limits were wider in the nasal visual field compared to the temporal visual field. The mean difference for both J_0_ and J_45_ were within 0.15 D and the 95% limits of agreement within 0.90 D across the horizontal visual field.

**Conclusion:**

Defocus components were similar between the Shin-Nippon autorefractor and the COAS-HD VR aberrometer with a 2.5-mm pupil for most visual field angles. However, there was a significant difference in defocus component between the Shin-Nippon autorefractor and the COAS-HD VR aberrometer with a 5.0-mm pupil, wherein the autorefractor measured more hyperopia. The astigmatic components J_0_ and J_45_ were similar between instruments for both central and peripheral refraction.

## Introduction

Cycloplegic autorefraction is a widely used technique to obtain objective refraction measures in clinical practice and research studies. This technique is considered reliable and accurate in the pediatric age.^1^ Furthermore, it is faster and relies less on the child's ability to cooperate than subjective refraction. Cycloplegic autorefraction is also the gold standard in epidemiological studies involving young children.^2^ Researchers are often interested in peripheral refraction because it provides information about peripheral defocus and the ocular shape.^3^^,^^4^ Objective central and peripheral refraction can be obtained with various instruments such as autorefractors,^5^, ^6^, ^7^, ^8^ photorefractors,^9^^,^^10^ and aberrometers.^6^^,^^11^^,^^12^ Measuring peripheral refraction has gained interest because it may be linked to myopia development.^6^^,^^13^, ^14^, ^15^, ^16^ Various clinical trials evaluating optical devices for myopia management in children, include measurements of peripheral refraction in their measurement protocol.^17^^,^^18^ Therefore, there is a need for research about reliability of peripheral refraction obtained in children.

Currently available instruments that measure objective refraction rely on different measurement principles. A commonly used instrument is the Shin-Nippon autorefractor (also marketed as Grand Seiko) that relies on classical measurement principles to measure refractive error.^19^^,^^20^ Innovations in technology have allowed the development of instruments based on wavefront analysis that measures the refractive state of the human eye.^21^ The Complete Ophthalmic Analysis System (COAS-HD VR aberrometer, Wavefront sciences, Albuquerque, NM) is one such instrument that uses multiple data points in the pupil to determine refraction from Zernike polynomials.^5^^,^^6^^,^^11^ Because instruments relying on classical and modern technology are used interchangeably, it is critical to investigate whether they provide similar measurements.

Few studies have compared central refraction values in children obtained with autorefraction and with aberrometry. A study conducted from McCullough et al.^11^ compared the IRX3 aberrometer (using 5-mm pupil diameter) with the Shin-Nippon SRW-5000 autorefractor. The study concluded that the difference in spherical equivalent refraction (SER) produced by the two instruments was close to 0.25 D. Similar findings were reported by Martinez et al., that compared the COAS G200 aberrometer (using 5-mm pupil diameter) with the Canon RK-F1 autorefractor. A systematic difference of, for example, 0.25 D between two instruments may lead to overestimation or underestimation of prevalence of refractive errors, such as myopia.

There is currently limited information about the validity of peripheral refraction obtained with open view autorefractors or with aberrometers in children. Specifically, there are no studies comparing peripheral refraction in children when using the Shin-Nippon autorefractor and the COAS-HD VR aberrometer. Children are expected to have more variable fixation (e.g. more square wave jerks)^22^ and may have difficulties to follow instructions during measurements.^23^ Given the differences in obtaining the measurement, results obtained in adults may not be directly applicable to children. The aim of this study was to compare central and peripheral refraction in children using the open view autorefractor Shin-Nippon NVision-K 5001 and the open view COAS-HD VR aberrometer. We hypothesize that different measurement principles can lead to significant differences in refraction between instruments.

## Methods

### Participants

Participants were recruited for a longitudinal study investigating the prevalence of refractive errors in Swedish schoolchildren. Details of the study protocol are available in our previous publication.^24^ For the current study, assuming 90% power, 95% confidence to a difference of 0.50 D between the two instruments - the required sample was 121 participants. Participants having any history of ocular pathology or surgery were excluded from the study. The research was approved by the regional ethics committee in Linköping (ref.  2018/423–31), and the study was conducted under the tenets of the Declaration of Helsinki. Written informed consent was obtained from the parent or legal guardian and written assent from all participants.

### Instruments

The Shin-Nippon NVision-K 5001 autorefractor uses three ring segments of infrared light (λ= 840 nm) that has a diameter of 2.32 mm. Therefore, the required minimum pupil size for measurement is 2.32 mm. Even though participants may have a larger pupil, the Shin-Nippon autorefractor always computes refraction by analyzing 2.32 mm image of the ring segments. A more detailed description of the Shin-Nippon autorefractor is given in Davies et al.^7^

The open-field COAS-HD VR aberrometer (Wavefront sciences, Albuquerque, NM) measures lower and higher-order monochromatic aberrations based on the Hartmann-Shack principle.^21^ It uses an infrared light source (λ= 840 nm) and an 83 × 62 array of lenslets with 108 µm in diameter. With this configuration, the device collects 766 samples from separated points distributed over a diameter of 5-mm. When the pupil diameter is smaller, the number of samples reduces, but the accuracy of the points measured is not compromised. A more detailed description of the COAS-HD VR aberrometer is provided in our previous paper.^25^

In both instruments, the vertex distance used to calculate refraction was set at the spectacle plane of 12-mm. Both instruments measure refractive error using infra-red light (840 nm) which then were converted to obtain refraction in the visible wavelength. The Shin-Nippon autorefractor converts to a wavelength of 547.06 nm (personal communication with Rexxam Co., Ltd) which was matched in the COAS-HD VR aberrometer.^26^ Also, only lower-order aberrations were used for comparison with the autorefractor. It was necessary to use the formulas given below to compute values of the M, J_0_, and J_45_ for the COAS-HD VR aberrometer.

$$M=\frac{-4\sqrt{3}\times C_{2}^{0}}{r^{2}}$$

$$J_{0}=\frac{-2\sqrt{6}\times C_{2}^{2}}{r^{2}}$$

$$J_{45}=\frac{-2\sqrt{6}\times C_{2}^{-2}}{r^{2}}$$

(*C^0^_2_ = defocus, C^2^_2_ = regular astigmatism, C^2^_2_ = irregular astigmatism, and r^2^ = radius of pupil diameter*). In addition, to measure the agreement between the Shin-Nippon autorefractor and the COAS- HD VR aberrometer, it is appropriate to match the pupil diameter for analysis.^5^^,^^27^ This approach is ideal for evaluating agreements between instruments but would not reflect a typical clinical scenario.^28^ Since the average photopic pupil diameter in this population was 4.3 ± 0.8 mm (range.  2.5 to 6.0 mm), a 5 mm pupil analysis diameter was also used. Previous studies have also compared two different pupil analysis diameters from aberrometers to autorefractor measurements.^5^^,^^28^^,^^29^

### Procedure

**Fig. 1:**
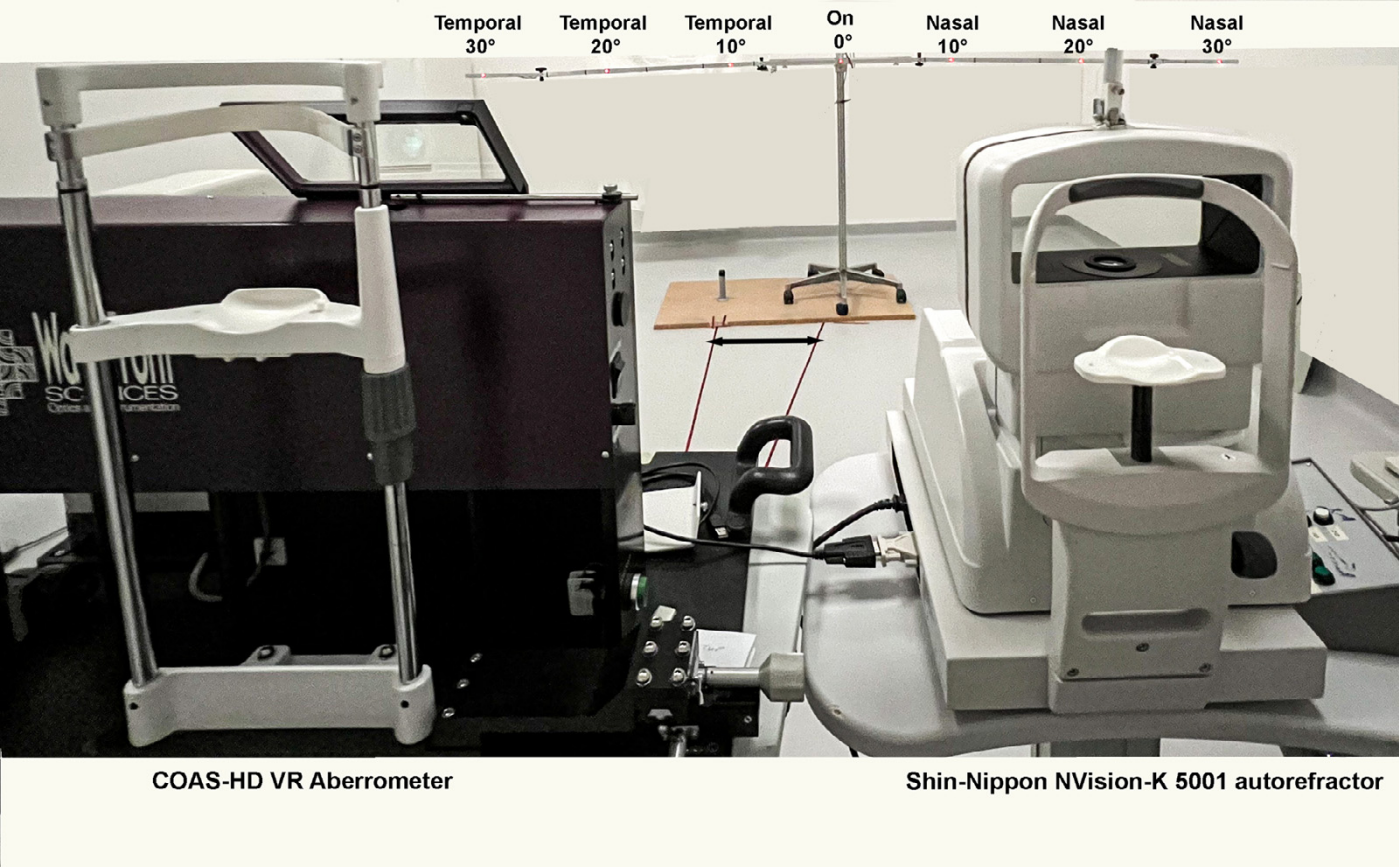
Shows experimental setup that was used to measure central and peripheral refraction in children. The participants fixated at the red-light emitting diodes that was located at 3 meters. The fixation target was moved and centered based on which instrument was used to measure the refraction (double arrowhead). Measurements were performed first on-axis at 0°,then in the temporal visual field and finally in the nasal visual field.

Prior to cycloplegia, participants underwent a complete eye examination that included visual acuity measurement and subjective refraction. Cycloplegia was induced by instilling two drops of 1.0% cyclopentolate hydrochloride in both eyes. The mean photopic pupil diameter pre-cyclopentolate was 4.3 mm (SD=0.8). Post cyclopentolate, the mean pupil diameter was 7.7 mm (SD=0.6). Three consecutive measurements of the central and peripheral refraction of the right eye were randomly obtained, either with the COAS-HD VR aberrometer or with the Shin-Nippon autorefractor. A single examiner (PD) performed all measurements. During measurements, participants were instructed to look at a series of fixation targets placed at a 3-meter distance (Figure 1). We have used this setup in our earlier studies, as 3 m simulates a distance viewing with minimal accommodation exerted before cycloplegia.^25^^,^^30^, ^31^, ^32^ Fixation targets were seven light emitting diodes (λ= 635 nm), that were evenly spaced (in 10° steps) along a semicircle starting at 30° nasal visual field and ending at 30° temporal visual field. Participants were instructed to fixate the target when it was switched on. Measurements were performed first on-axis at 0°, then temporally and finally nasally. We did not randomize the order of obtaining refraction from different eccentricities to avoid confusion during data extraction.

### Data analysis

The three measurements obtained from both instruments were converted into vector components M, J_0_, and J_45_.^33^ The coefficient of repeatability (CR) was determined by (i) calculating the variance of three repeated measurements and (ii) computing the square root of the mean variance for all participants, producing the within-subject standard deviation, (iii) within-subject standard deviation was multiplied by 1.96 to obtain the measurement error or CR. Lower absolute values of CR correspond to more repeatable measurements. The relative peripheral refraction was calculated as the spherical equivalent of the mean on-axis refraction subtracted from the spherical equivalent of the mean off-axis refraction for all six visual field angles. To further investigate the agreement between the two instruments, spherical aberrations were calculated for both 2.5-mm pupil and 5.0-mm pupil by COAS HD-VR aberrometer using the instruments’ inbuilt “Seidel sphere” method (see Appendix 1).^26^^,^^34^ Statistical analysis was performed with IBM SPSS (v26 for Windows, IBM Corp Armonk, NY). The agreement between both instruments was evaluated using Bland-Altman analysis, plotted with the GraphPad Prism (v9 for Windows, GraphPad, San Diego, CA).

## Results

One hundred and twenty-three children aged 8 to 16 years having best-corrected acuity better than 6/7.5 (logMAR 0.1) participated in this study. The mean age of the participants was 12.0 years (SD=2.5 years), 67 were females (54.5%) and 56 were males (45.5%). The mean (SD) central refractive error determined by the Shin-Nippon autorefractor was +0.70 D (SD=1.23 D), range −3.29 D to +5.62 D. The mean central refractive error determined by the COAS-HD VR aberrometer using a 2.5-mm pupil was +0.39 D (SD=1.25 D), range −3.73 D to +5.11 D and when using a 5-mm pupil was +0.19 D (SD=1.20 D), range −3.92 D to +4.51 D. Subjective refraction is reported in Appendix 1.

### Coefficient of repeatability

The CR for central refraction for the Shin-Nippon was 0.29 D for defocus, 0.21 D for J_0_, and 0.18 D for J_45_. For the COAS-HD VR and a 2.5-mm pupil, the CR was 0.24 D for defocus, 0.21 D for J_0_, and 0.14 D for J_45_. CR can be considered similar between Shin-Nippon autorefractor and COAS-HD VR using a 2.5-mm pupil for all three refraction components. CR was 0.12 D for defocus, 0.05 D for J_0_, and 0.07 D for J_45_ with the COAS-HD VR with a 5-mm pupil for all three refraction components. The most repeatable measurements were obtained with the COAS-HD VR for the 5-mm pupil diameter. Table 1 shows CR for both Shin-Nippon autorefractor and COAS-HD VR aberrometer in all seven eccentricities.

**Table 1:** shows coefficient of repeatability for central and peripheral refraction from Shin-Nippon NVision K-5001 autorefractor and COAS-HD VR aberrometer for two pupil sizes.

Refraction	Instrument	Central	Nasal visual field			Temporal visual field		
		0°	10°	20°	30°	10°	20°	30°
M	Shin-Nippon	0.29	0.32	0.35	0.43	0.26	0.30	0.29
	COAS 2.5-mm	0.24	0.26	0.31	0.25	0.23	0.21	0.25
	COAS 5.0-mm	0.12	0.15	0.20	0.15	0.12	0.13	0.20
								
J_0_	Shin-Nippon	0.21	0.30	0.26	0.33	0.25	0.29	0.26
	COAS 2.5-mm	0.21	0.16	0.25	0.22	0.18	0.12	0.22
	COAS 5.0-mm	0.05	0.12	0.20	0.17	0.08	0.09	0.11
								
J_45_	Shin-Nippon	0.18	0.21	0.14	0.20	0.22	0.28	0.17
	COAS 2.5-mm	0.14	0.21	0.16	0.16	0.13	0.12	0.14
	COAS 5.0-mm	0.07	0.06	0.08	0.11	0.06	0.06	0.08
								

### Central and peripheral refraction

**Fig. 2:**
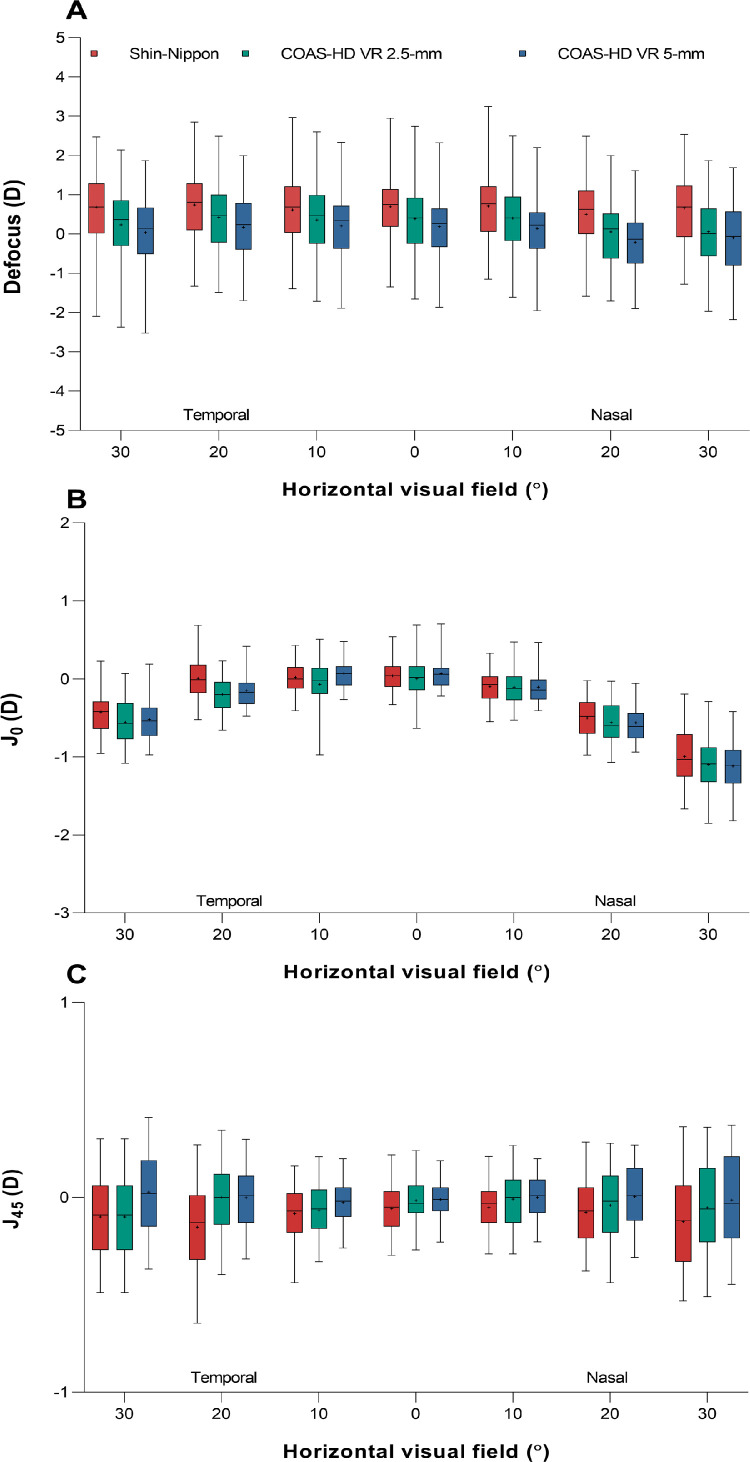
Box and whisker plot showing the distribution of refractive error for the three refractive components, A) defocus, B) J_0_, and C) J_45_ across the horizontal visual field for Shin-Nippon autorefractor and COAS-HD VR aberrometer (for both 2.5-mm and 5-mm). Boxes show the 25th to the 75th percentile and whiskers range from the 5th to 95th percentile, a “+” sign indicates the mean and the horizontal line represents the median.

Fig. 2 shows the three refractive components, defocus (panel A), J_0_ (panel B) and J_45_ (panel C) across the horizontal visual field for the Shin-Nippon autorefractor and the COAS-HD VR aberrometer (for both 2.5 and 5-mm pupil diameter).

A mixed-design ANOVA revealed a statistically significant difference for defocus between the two instruments, F (2, 366) = 8.32, MSE= 78.62, *p* < .001. A post-hoc Tukey revealed that the defocus was significantly different in Shin-Nippon autorefractor when compared to COAS-HD VR aberrometer for both the 2.5-mm (*p* = .028) and 5-mm pupil diameter (*p* < .001). Defocus also changed significantly across eccentricity with F (2.44, 891.06) = 33.47, MSE= 14.20, *p* < .001. The interaction between instrument and eccentricity was significant, F (4.87, 891.06) = 3.94, MSE= 1.67, *p* = .02. We investigated the interaction by performing simple effects pairwise comparisons with Bonferroni adjustments. This confirmed the main effects, that is, defocus from the Shin-Nippon was significantly different across the entire horizontal visual field compared to the COAS-HD VR 5-mm pupil diameter. For 2.5-mm pupil diameter differences were statistically significant only at temporal 30°, nasal 20° and 30°.

Measurements of regular astigmatism (J_0_) differed significantly between the two instruments with F (2, 366) = 8.32, MSE= 1.79, *p* = .019. A post-hoc Tukey revealed that J_0_ from the Shin-Nippon autorefractor was significantly different from the COAS-HD VR aberrometer for 2.5-mm pupil (*p* = .028) but not for 5-mm pupil (*p* = .25). J_0_ also changed significantly across eccentricity with F (2.92, 1067.17) = 1190.45, MSE= 122.44, *p* < .001 and revealed an expected temporal-nasal asymmetry.^30^^,^^35^^,^^36^ The interaction between instrument and eccentricity was significant, F (5.83, 1067.17) = 1190.45, MSE= 122.44, *p* < .001. Simple effects analyses with Bonferroni adjustments indicated statistically significant difference between Shin-Nippon and COAS-HD VR (for both pupil diameters) for J_0_ only for temporal 20° and 30°.

Irregular astigmatism (J_45_) differed significantly between the two instruments. A post-hoc Tukey revealed that J_45_ obtained with the Shin-Nippon autorefractor was significantly different from J_45_ obtained with the COAS-HD VR aberrometer for 2.5-mm (*p* < .001) and for 5-mm pupil (*p* < .001). J_45_ also changed significantly across eccentricity with F (1.87, 683.44) = 5.37, MSE= 0.52, *p* = .006. The interaction between instrument and eccentricity was significant, F (3.74, 683.44) = 5.37, MSE= 0.37, *p* = .006. Simple effects analyses with Bonferroni adjustments indicated that J_45_ from the Shin-Nippon was significantly different from the COAS-HD VR 5-mm pupil across the entire horizontal visual field. For the 2.5-mm pupil, differences were statistically significant only at temporal 20° and 30°.

### Agreement between instruments for central and peripheral refraction

**Fig. 3:**
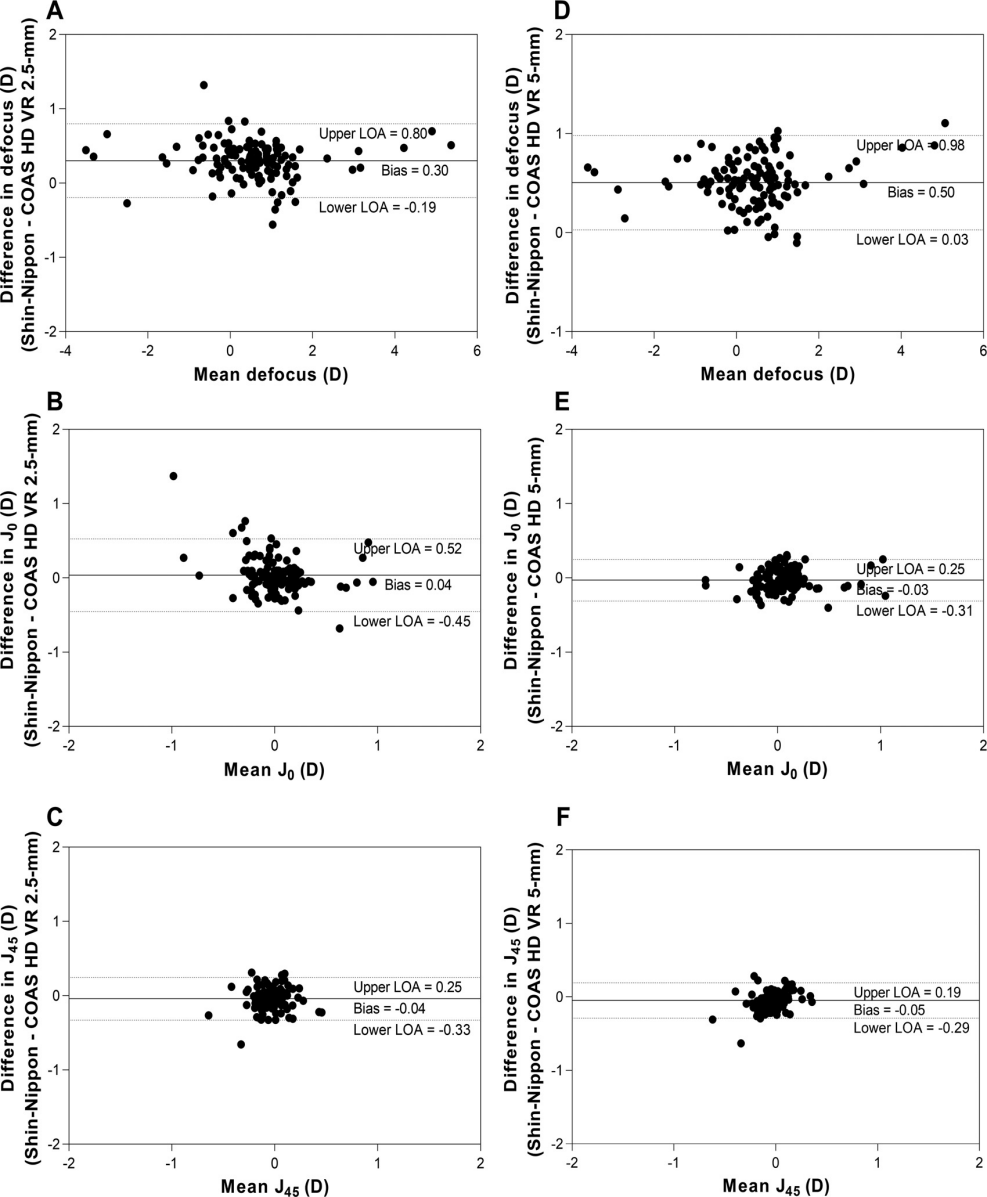
Bland- Altman plots showing agreement between Shin-Nippon NVision-K 5001 autorefractor and COAS-HD VR aberrometer for central refraction. X-axes represent the mean estimate for both methods.  Y-axes represent the estimate difference between the two instruments (Shin-Nippon – COAS HD VR). The solid line indicates the bias, and the 95% upper and lower limits are indicated by the dotted lines. Panels A, B and C show the agreement between Shin-Nippon and COAS-HD VR 2.5-mm, and panels D, E and F show the agreement between Shin-Nippon and COAS-HD VR 5-mm for all three refractive components.

Fig. 3 shows Bland-Altman plots for agreement between Shin-Nippon and COAS-HD VR aberrometer for central refraction. Panels A, B and C show the agreement between Shin-Nippon and COAS-HD VR 2.5-mm pupil, and panels D, E and F show the agreement between Shin-Nippon and COAS-HD VR 5-mm pupil for the three refractive components.

The mean differences (bias) for defocus between Shin-Nippon and COAS-HD VR were 0.30 D for 2.5-mm pupil (panel A) and 0.50 D for 5-mm pupil (panel D). The bias for both J_0_ and J_45_ were close to zero for both pupil diameters (panel B, C, E and F). In Appendix 1, we also provide extra analysis of these measurements. Peripheral refraction agreements between the two instruments for the two different pupil sizes are summarized in Table 2.

**Table 2:** Bias and limits of agreement between Shin-Nippon autorefractor and COAS-HD VR aberrometer along the horizontal visual field.

	Nasal visual field			Temporal visual field		
2.5-mm pupil	10°	20°	30°	10°	20°	30°
Defocus M Bias* ± SD. Repeatability (1.96 x SD) Upper LoA (CI) Lower LoA (CI)	0.31 ± 0.41 0.80 1.12 (1.00 to 1.26) −0.50 (−0.64 to −0.39)	0.45 ± 0.43 0.84 1.29 (1.17 to 1.43) −0.39 (−0.54 to −0.28)	0.60 ± 0.55 1.10 1.68 (1.53 to 1.87) −0.47 (−0.66 to −0.32)	0.25 ± 0.32 0.62 0.88 (0.80 to 0.99) −0.38 (−0.49 to −0.29)	0.32 ± 0.26 0.51 0.83 (0,76 to 0.92) −0.19 (−0.28 to −0.12)	0.45 ± 0.39 0.76 1.21 (1.11 to 1.35) −0.32 (−0.46 to −0.22)
J_0_ Bias* ± SD. Repeatability (1.96 x SD) Upper LoA (CI) Lower LoA (CI)	0.01± 0.22 0.43 (0.37 to 0.51)−0.42 (−0.49 to −0.36)	0.10 ± 0.24 0.47 0.53 (0.46 to 0.61) −0.40 (−0.49 to −0.34)	0.11 ± 0.46 0.90 1.01 (0.89 to 1.17) −0.80 (−0.96 to −0.68)	0.09 ± 0.35 0.67 0.78 (0.68 to 0.90) −0.60 (−0.71 to −0.50)	0.21 ± 0.25 0.49 0.70 (0.63 to 0.78) −0.28 (−0.37 to −0.22)	0.13 ± 0.26 0.51 0.63 (0.56 to 0.72) −0.37 (−0.46 to −0.30)
J_45_ Bias* ± SD. Repeatability (1.96 x SD) Upper LoA (CI) Lower LoA (CI)	−0.04 ± 0.17 0.33 0.28 (0.24 to 0.34) −0.37 (−0.43 to −0.21)	−0.04 ± 0.17 0.33 0.29 (0.45 to 0.35) −0.37 (−0.42 to −0.32)	−0.10 ± 0.18 0.35 0.29 (0.24 to 0.35) −0.43 (−0.49 to −0.37)	−0.02 ± 0.18 0.35 0.33 (0.30 to 0.39) −0.36 (−−0.42 to −0.31)	−0.15 ± 0.23 0.45 0.29 (0.23 to 0.37) −0.60 (−0.67 to −0.53)	−0.15 ± 0.17 0.33 0.18 (0.14 to 0.24) −0.49 (−0.54 to −0.44)
5-mm pupil						
Defocus M Bias* ± SD. Repeatability (1.96 x SD) Upper LoA (CI) Lower LoA (CI)	0.57 ± 0.45 0.88 1.46 (1.34 to 1.61) −0.32 (−0.47 to −0.19)	0.71 ± 0.41 0.80 1.52 (1.41 to 1.70) −0.10 (−0.23 to 0.02)	0.76 ± 0.50 0.98 1.75 (1.61 to 1.92) −0.22 (−0.40 to −0.10)	0.41 ± 0.29 0.57 0.98 (0.90 to 1.10) −0.15 (−0.26 to −0.10)	0.57 ± 0.29 0.57 1.15 (1.10 to 1.25) −0.01 (−0.11 to 0.10)	0.64±0.37 0.73 1.36 (1.26 to 1,49) −0.10 (−0.20 to 0.10)
J_0_ Bias* ± SD. Repeatability (1.96 x SD) Upper LoA (CI) Lower LoA (CI)	0.01 ± 0.18 0.350.36 (0.3 to 0.43)−0.35 (−0.41 to −0.30)	0.06 ± 0.20 0.39 0.46 (0.40 to 0.52) −0.33 (−0.40 to −0.28)	0.12 ± 0.34 0.67 0.79 (0.70 to 0.91) −0.54 (−0–66 to −0.45)	−0.10 ± 0.18 0.35 0.29 (0.25 to 0.35) −0.40 (−0.46 to−0.34)	0.16 ± 0.22 0.43 0.58 (0.52 to 0.65) −0.34 (−0.34 to −0.20)	0.10 ± 0.21 0.41 0.50 (0.44 to 0.57) −0.31 (−0.39 to −0.26)
J_45_ Bias* ± SD. Repeatability (1.96 x SD) Upper LoA (CI) Lower LoA (CI)	−0.05 ± 0.12 0.24 0.19 (0.16 to 0.23) −0.29 (−0.33 to −0.26)	−0.08 ± 0.14 0.27 0.19 (0.15 to 0.24) −0.35 (−0.40 to −0.32)	−0.11 ± 0.17 0.33 0.21 (0.17 to 0.27) −0.43 (−0.49 to −0.39	−0.06 ± 0.15 0.29 0.24 (0.20 to 0.30) −0.36 (−0.41 to −0.32	−0.15 ± 0.21 0.41 0.26 (0.20 to 0.33) −0.56 (−0.63 to −0.50)	−0.13 ± 0.15 0.29 0.17 (0.13 to 0.22) −0.42 (−0.48 to −0.38)

The mean differences with repeatability (1.95 XSD) and the 95% upper and lower limits of agreement (95% confidence interval) between Shin-Nippon and COAS-HD VR for two different pupil sizes (2.5-mm and 5-mm) along the horizontal visual field.

The 95% limits of agreement for defocus across horizontal visual field were within 1.10 D for 2.5-mm pupil and within 0.98 D for 5-mm pupil. The limits were wider in the nasal visual field compared to the temporal visual field for both pupil sizes. The 95% limits of agreement for the J_0_ across horizontal visual field were within 0.90 D for 2.5-mm pupil and within 0.67 D for 5-mm pupil. The limits were within 0.50 D across the horizontal visual field except for the 30° nasal visual field for both pupil sizes. The 95% limits of agreement for the J_45_ across horizontal visual field were within 0.45 D for 2.5-mm pupil and within 0.41 D for 5-mm pupil. The limits were similar across the eccentricity except for the 20° temporal field in both pupil sizes.

**Fig. 4:**
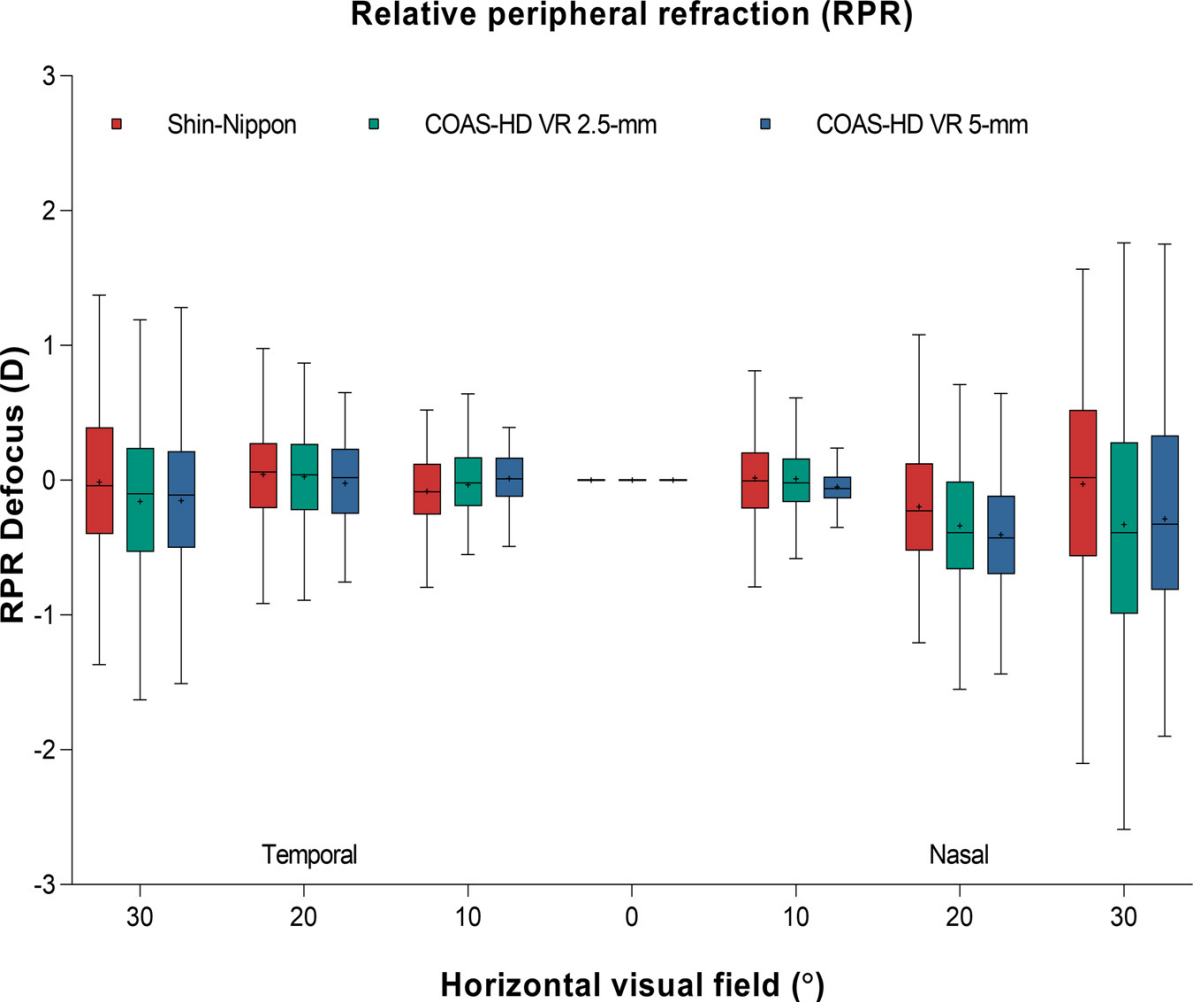
Box and whisker plot showing the distribution of refractive error of the relative peripheral refraction across the horizontal visual field for Shin-Nippon autorefractor and COAS-HD VR aberrometer (for both 2.5-mm and 5-mm). Boxes show the 25th to the 75th percentile and whiskers range from the 5th to 95th percentile, a “+” sign indicates the mean and the horizontal line represents the median.

Fig. 4 shows the relative peripheral refraction across the horizontal visual field for Shin-Nippon autorefractor and COAS-HD VR aberrometer (for both 2.5-mm and 5-mm pupil).

## Discussion

In this study, we compared central and peripheral refraction in children given by two open view instruments. We also evaluated the coefficient of repeatability of both instruments for central and peripheral refraction. We found that the best repeatability was obtained with the COAS-HD VR with a 5-mm pupil. Repeatability was worse for the Shin-Nippon autorefractor and the COAS-HD VR with a 2.5-mm pupil for both central and peripheral refraction. For central refraction, the Shin-Nippon autorefractor was 0.30 D more hyperopic than the COAS-HD VR aberrometer when using a 2.5 mm pupil and 0.50 D more hyperopic than the COAS-HD VR when using a 5 mm pupil. As given in Appendix 1, measurements from the COAS-HD VR considering a 5 mm pupil were the most consistent with subjective refraction. For both pupil diameters, the 95% limits of agreement were approximately 0.50 D for central refraction with wider limits in the nasal visual field compared with the temporal visual field for both sizes of pupil tested.

### Refraction repeatability

The Shin-Nippon autorefractor showed an average variability of 0.29 D for defocus between measurements for central refraction. A previous study that investigated intersession repeatability using the Shin-Nippon NVision-K 5001 autorefractor showed a CR of 0.10 D for defocus in adults.^7^ Another study involving children aged 5 to 8 years found a CR of 0.38 D for a Shin-Nippon SRW-5000 autorefractor.^37^ A study by Lee and Cho^38^ showed that the CR of the Shin-Nippon N Vision-K 5001 autorefractor in children aged 6 to 9 years was 0.41 D for central refraction, which was higher than the value reported in our study. The CR for the Shin-Nippon autorefractor worsened with increasing eccentricity. Worse repeatability may be caused by a combination of variable fixation, operator misalignment between measurements, and a smaller ring image.^29^ The slight misalignment in fixation between each measurement would lead to slightly different refraction obtained when the analysis is performed for smaller pupil or ring image. Our findings of better repeatability with the COAS-HD VR aberrometer than with the Shin-Nippon autorefractor support the idea that sampling over an area of 5-mm produce more repeatable results, particularly for peripheral refraction. Analyzing larger areas of the pupil might also be beneficial because measurements may be less sensitive to the effect of fixation.^22^

### Comparison between instruments for central refraction

The central defocus component in our study showed that the Shin-Nippon autorefractor measured more hyperopic central refraction than the COAS-HD VR aberrometer for both the pupil diameters analyzed, as well as the subjective refraction before cycloplegia (see Appendix 1). These results are in line with other authors comparing refractive values from autorefractors and aberrometers.^7^^,^^16^^,^^39^^,^^40^ Others^11^^,^^12^ have found a systematic difference of 0.25 D between these two types of instruments when measuring refraction in children, where autorefractor has always produced a more hyperopic refraction.^5^ In a similar comparison, McCullough et al.^11^ reported that the IRX3 aberrometer measured more hyperopic values than the Shin-Nippon SRW-5000 autorefractor in children aged 9 to 10 years. In contrast, for ages 15 and 16-years the authors reported more hyperopic results with the autorefractor than with the aberrometer. Finding from McCullough et al. were inconsistent. The effect of age is probably related to having difficulties maintaining fixation during measurements. Greater myopic refraction provided by the COAS-HD VR aberrometer with a 5-mm pupil may be due to the use of data points from the entire pupil^41^ as opposed to the smaller ring image of the Shin-Nippon autorefractor.

In line with previous studies, the 95% limits of agreement between the compared instruments for the J_0_ and J_45_ were within 0.50 D for central refraction.^11^^,^^12^ A study by Nguyen et al. compared the Discovery System aberrometer and Grand Seiko WAM-5500 autorefractor for two pupil sizes (3-mm and 6-mm pupil diameter)^12^ and also found statistically significant differences between the two instruments for J_0_ and J_45_. Even though there was a statistically significant difference, from a clinical perspective the difference can be considered negligible.

### Comparison between instruments for peripheral refraction

The Shin-Nippon autorefractor measured relatively more hyperopic peripheral defocus than the COAS-HD VR aberrometer. This finding is in agreement with Berntsen et al., who also reported that the Grand-Seiko WR-5100 K autorefractor produced more hyperopic refraction than the COAS-HD VR aberrometer in young adults.^29^ The mean difference between the Shin-Nippon autorefractor and the COAS-HD VR aberrometer reduces when spherical aberration is included to compute defocus in the aberrometer (see Appendix 1). The Shin-Nippon autorefractor measured more hyperopic defocus than the aberrometer across the 7 points tested in the horizontal visual field, but that did not affect the difference between central and peripheral refraction - the so called relative peripheral refraction. Therefore, both instruments showed a similar trend in the relative peripheral refraction and can be used to obtain peripheral refraction in children despite poor repeatability with the Shin-Nippon autorefractor.

### Limitations

Participants in this study were instructed to turn their eyes at the fixation target. The extraocular muscles might distort eye shape and thereby alter peripheral refraction with eye turns. However, previous studies reported no significant difference between peripheral refraction made with the eye turn and the head turn method.^42^^,^^43^ Therefore, small residual eye turns are unlikely to have significantly influenced peripheral refraction and its repeatability.

In conclusion, clinicians and researchers should be aware of the difference in measurements produced by the Shin-Nippon autorefractor and the COAS-HD VR aberrometer. This study points to the sources of the systematic difference between the two instruments that affect defocus values. Caution must be taken when reporting results from different instruments or when comparing results of studies conducted with instruments that rely on different measurement principles.

## Appendix Supplementary materials

Image, application 1
