# Supplementary material for: Comparison of an open view autorefractor with an open view aberrometer in determining peripheral refraction in children
Source: J Optom. 2022 Jan 10;16(1):20–9. doi: 10.1016/j.optom.2021.12.002 (PMC9811364; doi:10.1016/j.optom.2021.12.002)
Supplement: Supplementary file 1 [file mmc1.zip › Appendix Table A1.docx]

Appendix table A1: Bias and limits of agreement between Shin-Nippon autorefractor and COAS-HD VR aberrometer (Seidel sphere) for defocus along the horizontal visual field.

|  | Central | Nasal visual field | | | | Temporal visual field | | |
| --- | --- | --- | --- | --- | --- | --- | --- | --- |
| 2.5-mm pupil  Defocus Seidel M | 0° | 10° | 20° | 30° | 10° | | 20° | 30° |
| Bias^*^ ± SD:  1.96 x SD  Upper LoA  Lower LoA | 0.31 ± 0.48  0.94  1.25  −0.63 | 0.14 ± 0.34  0.66  0.80  −0.52 | 0.36 ± 0.47  0.92  1.28  −0.56 | 0.52 ± 0.51  0.99  1.51  −0.47 | 0.27 ± 0.47  0.92  1.19  −0.65 | | 0.25 ± 0.39  0.76  1.01  −0.51 | 0.40 ± 0.48  0.94  1.34  −0.54 |
| 5-mm pupil  Defocus Seidel M | 0° | 10° | 20° | 30° | 10° | | 20° | 30° |
| Bias^*^ ± SD:  1.96 x SD  Upper LoA  Lower LoA | 0.19 ± 0.22  0.43  0.62  −0.24 | 0.22 ± 0.27  0.53  0.75  −0.31 | 0.41 ± 0.39  0.76  1.17  −0.35 | 0.55 ± 0.42  0.82  1.37  −0.27 | 0.12 ± 0.27  0.53  0.65  −0.41 | | 0.28 ± 0.25  0.49  0.77  −0.21 | 0.40 ± 0.37  0.73  1.13  −0.33 |
