# Supplementary material for: Comparison of an open view autorefractor with an open view aberrometer in determining peripheral refraction in children
Source: J Optom. 2022 Jan 10;16(1):20–9. doi: 10.1016/j.optom.2021.12.002 (PMC9811364; doi:10.1016/j.optom.2021.12.002)
Supplement: Supplementary file 1 [file mmc1.zip › Appendix 1.docx]

Supplementary results:

## *Comparison of central refraction between instruments and subjective refraction*

Supplementary figure 1 shows the difference in mean spherical equivalent central refraction between autorefraction (post cycloplegic) and subjective refraction (pre cycloplegic).

One-way repeated measures ANOVA indicated that there were statistically significant differences between subjective refraction and autorefraction (Shin-Nippon autorefractor and COAS-HD VR aberrometer 2.5-mm and 5-mm pupil) for defocus with F (3, 488) = 7.91, p<.001. Post hoc Tukey pairwise comparisons showed that there was a statistically significant difference between subjective refraction (0.007 D) and Shin-Nippon autorefractor (0.69 D) with p < .001, as well as subjective refraction and COAS-HD VR 2.5-mm pupil (0.39 D) with p=.046. There was no statistically significant difference between subjective refraction and COAS-HD VR 5.0-mm pupil (0.18 D) with p=.603. ANOVA showed no statistically significant difference between subjective refraction and autorefraction in determining the J_0_ astigmatism with F (3,488) = 1.29, p = 0.276. For the J_45_ astigmatism, ANOVA showed an overall significant difference between subjective refraction and autorefraction with F (3,488) = 2.87, p = .036 but a post hoc Tukey revealed that this difference was only significant between Shin-Nippon autorefractor and COAS-HD VR 5-mm pupil (p = .043) but not between subjective refraction and autorefraction.

Supplementary figure 2 shows Bland-Altman agreement for defocus (M) that demonstrates the bias and limits of agreement between subjective refraction and autorefraction. Mean pre-cycloplegic subjective refraction was in close agreement with COAS-HD VR 5-mm pupil measuring slightly hyperopic with a mean difference of 0.18 D. Both COAS-HD VR 2.5-mm pupil (0.39 D) and Shin-Nippon autorefractor (0.69 D) measured more hyperopic than subjective refraction. Nevertheless, the limits of agreement between subjective refraction and autorefraction from both instruments were wider due to underestimation of hyperopia prior to cycloplegia.

*Comparison of Shin-Nippon autorefractor defocus with COAS-HD VR seidel sphere*

Supplementary figure 3 shows the defocus across the horizontal visual field for the Shin-Nippon autorefractor and the COAS-HD VR aberrometer Seidel sphere (for both 2.5 and 5-mm pupil diameter). A mixed-design ANOVA showed that there was no statistically significant difference in defocus between the Shin-Nippon autorefractor and the COAS-HD VR aberrometer seidel sphere (defocus that includes primary spherical aberration), F (2, 366) = 2.92, MSE= 511.04, p = .055. There was statistically significant difference in defocus across eccentricity with F (2.74, 1002.68) = 28.98, MSE= 12.85, p < .001. The interaction between instrument and eccentricity was significant, F (5.48, 1002.68) = 4.53, MSE= 2.00, p = <.001. We investigated the interaction by performing simple effects pairwise comparisons with Bonferroni adjustments. We found that there was no significant difference in defocus between the Shin-Nippon autorefractor and the COAS-HD VR aberrometer (for both pupil sizes) in central and temporal visual field as well as nasal 10° (p > .05). However, there was statistically significant differences at nasal 20° and 30° between the Shin-Nippon autorefractor and the COAS-HD VR aberrometer (for both pupil sizes).

Supplementary table shows the bias and limits of agreement between Shin-Nippon autorefractor and COAS-HD VR aberrometer (Seidel sphere) for defocus along the horizontal visual field. The bias for COAS-HD VR with 2.5-mm pupil remains similar with or without inclusion of spherical aberration, due to impact of spherical aberration in influencing refraction is limited with smaller pupil diameter. COAS-HD VR with 5-mm pupil that includes spherical aberration clearly shows more hyperopic refraction both for central and peripheral refraction and closely agrees with the Shin-Nippon autorefractor (except for nasal 20° and 30°). The mean bias for central refraction between the Shin-Nippon autorefractor and COAS-HD VR aberrometer with 5-mm pupil also reduces from 0.50 D to 0.19 D when spherical aberration is included in defocus calculation. These results clearly show the influence of spherical aberration on lower order defocus if larger pupil analysis diameter is chosen in the aberrometer.
