# Supplementary figures and images for: Comparison of an open view autorefractor with an open view aberrometer in determining peripheral refraction in children
Source: J Optom. 2022 Jan 10;16(1):20–9. doi: 10.1016/j.optom.2021.12.002 (PMC9811364; doi:10.1016/j.optom.2021.12.002)

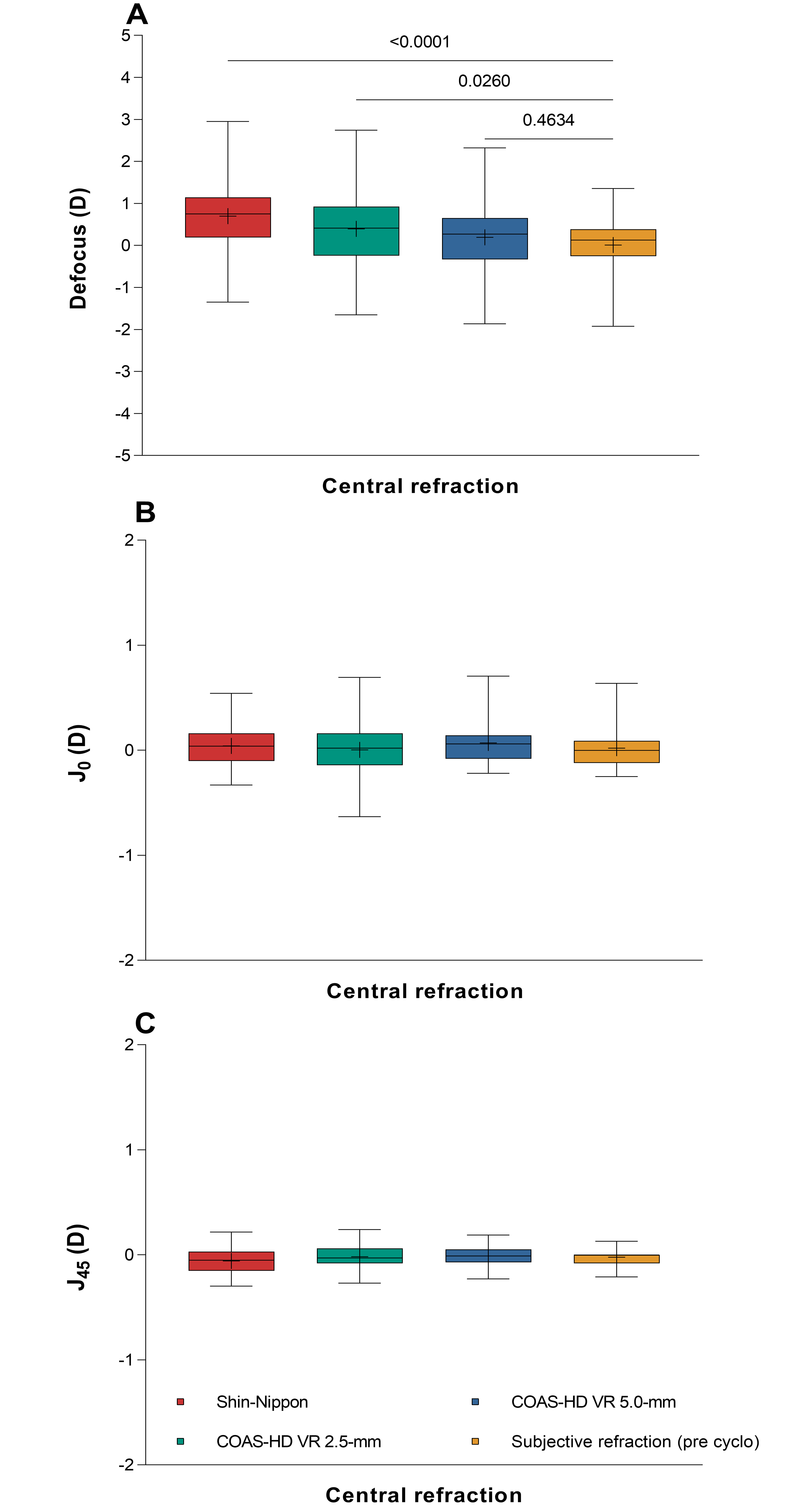

Supplement: Supplementary file 1 [file mmc1.zip › Appendix Figure A1.tif]

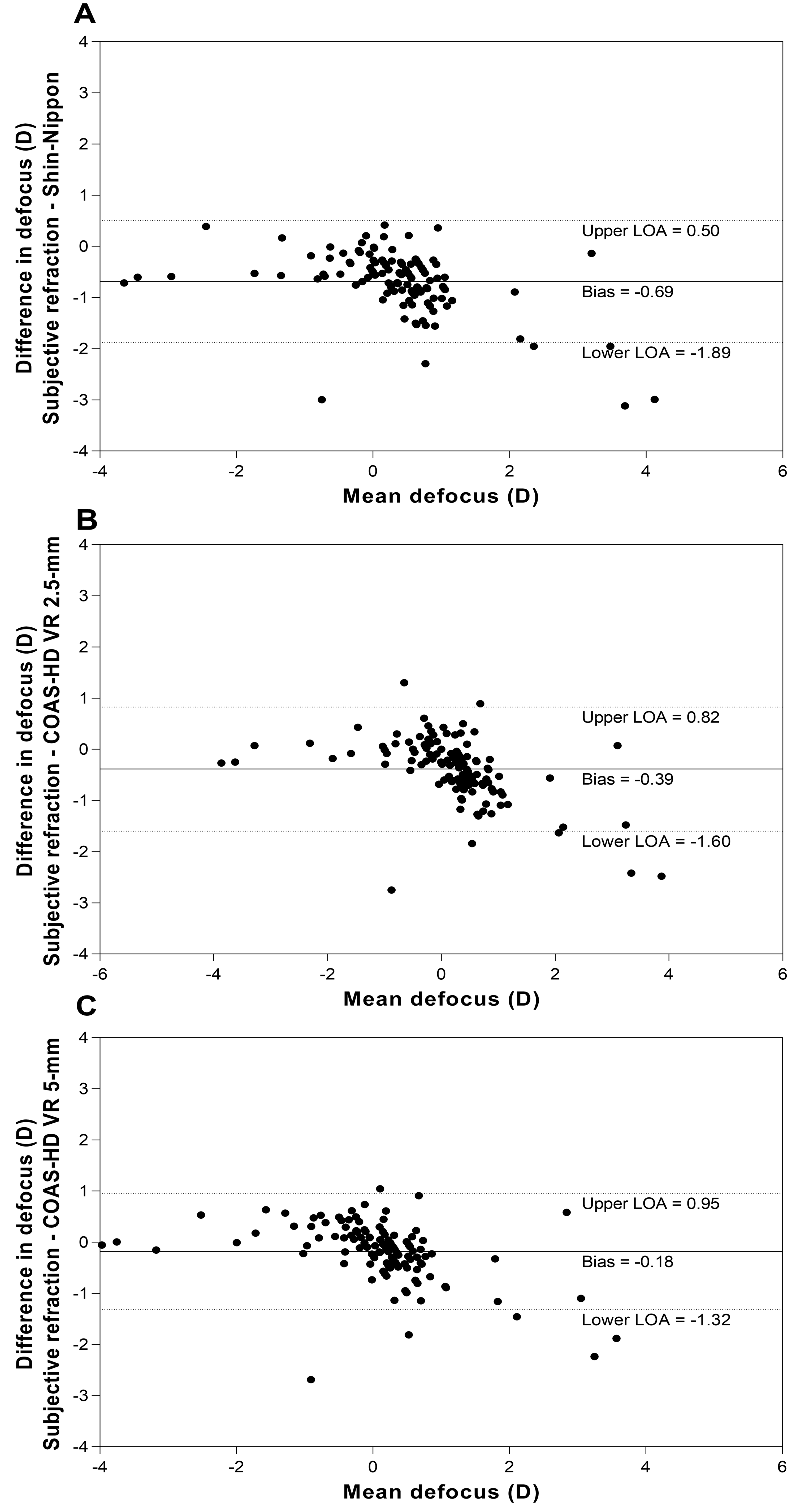

Supplement: Supplementary file 1 [file mmc1.zip › Appendix Figure A2.tif]

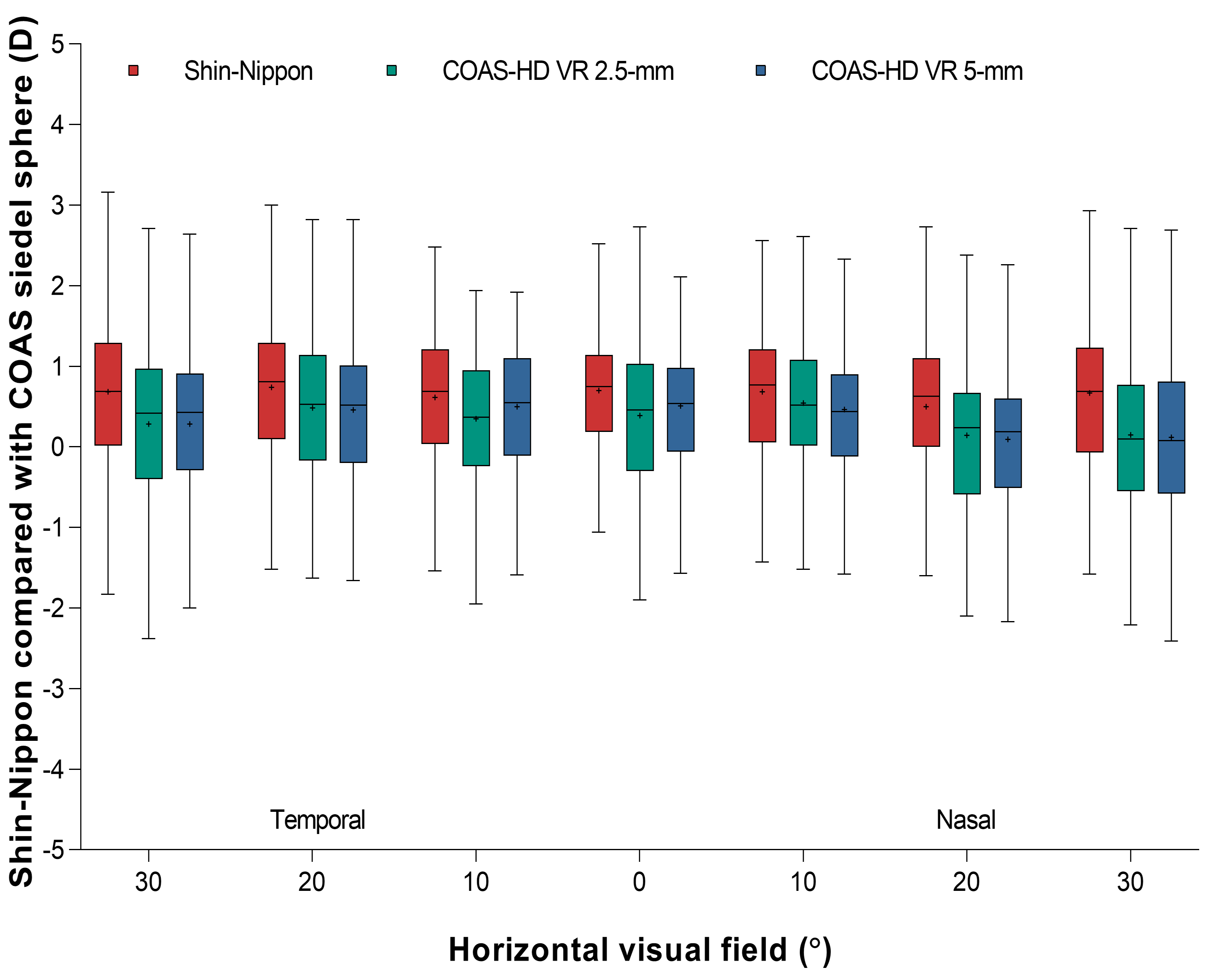

Supplement: Supplementary file 1 [file mmc1.zip › Appendix Figure A3.tif]
